# Supplementary material for: Fusarium oxysporum Casein Kinase 1, a Negative Regulator of the Plasma Membrane H+-ATPase Pma1, Is Required for Development and Pathogenicity
Source: J Fungi (Basel). 2022 Dec 15;8(12):1300. doi: 10.3390/jof8121300 (PMC9786551; doi:10.3390/jof8121300)
Supplement: Supplementary file 1 [file jof-08-01300-s001.zip › jof-2087380-supplementary.pdf]

Supplementary Table S1. List of primers used in this study. Lowercase nucleotides do not belong to the original sequence and were introduced to generate overlapping ends for fusion PCR reactions

Gene	Name	[Tm]_Sequence 5'→3'
hyg	HygG	[62]_CGTTGCAAGACCTGCCTGAA
	HygY	[62]_GGATGCCTCCGCTCGAAGTA
phleo	Leo	[62]_GCCACGAAGTGCACGCAGTT
	Phe-5	[62]_CGGAGCGGTCGAGTTCTGG
Casete *hyg/phleo*	gpdA15B	[62]_GGATCCCGAGACCTAATACAGCCCCT
	trpC8B	[62]_GGATCCAAACAAGTGTACCTGTGCATTC
ck1	Ck1-1	[62]_GAGGTAGGATTGGGTGCGAC
	Ck1-2	[62]_CACGGGAAGGGTTGAGAGAG
	Ck1-3	[62]_tttacccagaatgcacaggtacacttgttATTTAGGCGGCG AAGGCGGT
	Ck1-4	[62]_tggtcgttgtaggggctgtattaggtctcgTCATTGCTGCGG CGTTGTGC
	Ck1-5	[62]_TCATCGTGTCGTTTCTCAGCG
	Ck1-6	[62]_TCTGTTTCACCCCTTCGTCCA
	Ck1-Fwd	[62]_GGCAAGAAGATTGGAGAGGG
	Ck1-Rv	[62]_CTCGCCATCCTCAACCTCG

```
YCk1       ---MSMPIAS--TTLAVNNL-----------TNINGNANFNVQANKQLHHQAVDSPARS 43
YCk2       MSQVQSPLTATNSGLAVNNNTMNSQMPNRSNVRLVNGTLPPSLHVSSNLN---------- 50
CnCk1      ----------------------------MATTHVIG---------------------S 9
MoCk1      ------------------------------------------------------------ 0
FoCk1      ------------------------------------------------------------ 0
VdCK1      ------------------------------------------------------------ 0


YCk1       SMTATTAANSNSNSSRDDSTIVGLHYKIGKKIGEGSFGVLFEGTNMINGVPVAIKFEPRK 103
YCk2       HNTGNSSASYSGSQSRDDSTIVGLHYKIGKKIGEGSFGVLFEGTNMINGLPVAIKFEPRK 110
CnCk1      SN-PGGHLTSAGSHSSSSSNIVGVHYKVGKKIGEGSFGVIFEGTNLLNSQTVAIKFEPRK 68
MoCk1      -------------MASSSSNVVGVHYRVGKKIGEGSFGVIFEGTNLLNNQQVAIKFEPRK 47
FoCk1      -------------MASSSSNVVGVHYRVGKKIGEGSFGVIFEGTNLLNNQQVAIKFEPRK 47
VdCK1      -------------MASSSSNVVGVHYRVGKKIGEGSFGVIFEGTNLLNNQQVAIKFEPRK 47
                         : ..*.:**:**::***********:*****::*.  *********

YCk1       TEAPQLRDEYKTYKILNGTPNIPYAYYFGQEGLHNILVIDLLGPSLEDLFDWCGRKFSVK 163
YCk2       TEAPQLKDEYRTYKILAGTPGIPQEYYFGQEGLHNILVIDLLGPSLEDLFDWCGRRFSVK 170
CnCk1      SDAPQLRDEYRSYKILSGCLGIPQVYYFGQEGLHNILVIDLLGPSLEDLFDMCGRKFSVK 128
MoCk1      SDAPQLRDEYRTYKILVGCPGIPNVYYFGQEGLHNILVIDLLGPSLEDLFDHCNRRFTIK 107
FoCk1      SDAPQLRDEYRTYKILVGCPGIPNVYYFGQEGLHNILVIDLLGPSLEDLFDHCGRRFSIK 107
VdCK1      SDAPQLRDEYRTYKILVGCPGIPNVYYFGQEGLHNILVIDLLGPSLEDLFDHCGRRFTIK 107
           ::****:***::**** *  .**  ************************** *.*:*::*

YCk1       TVVQVAVQMITLIEDLHAHDLIYRDIKPDNFLIGRPGQPDANNIHLIDFGMAKQYRDPKT 223
YCk2       TVVQVAVQMITLIEDLHAHDLIYRDIKPDNFLIGRPGQPDANKVHLIDFGMAKQYRDPKT 230
CnCk1      TCCMTAKQMLSRVQTIHEKNLIYRDIKPDNFLIGRPSSKNANLIHVVDFGMAKQYRDPKT 188
MoCk1      TVVMVAKQMLSRVQTIHEKNLIYRDIKPDNFLIGRPGTKTANVIHVVDFGMAKQYRDPKS 167
FoCk1      TVVMVAKQMLSRVQTIHEKNLIYRDIKPDNFLIGRPGTKASSVIHVVDFGMAKQYRDPKT 167
VdCK1      TVVMVAKQMLSRVQTIHEKNLIYRDIKPDNFIIGRPGTKAANVIHVIDFGMAKQYRDPKT 167
           *   .* **:: :: :* ::***********:****.   :. :*::************:

YCk1       KQHIPYREKKSLSGTARYMSINTHLGREQSRRDDMEALGHVFFYFLRGHLPWQGLKAPNN 283
YCk2       KQHIPYREKKSLSGTARYMSINTHLGREQSRRDDMEAMGHVFFYFLRGQLPWQGLKAPNN 290
CnCk1      KQHIPYRERKSLSGTARYMSINTHLGREQSRRDDLEALGHVFFYFLRGGLPWQGLKAATN 248
MoCk1      KQHIPYRERKSLSGTARYMSINTHLGREQSRRDDLEALGHVFMYFLRGGLPWQGLKAATN 227
FoCk1      KQHIPYRERKSLSGTARYMSINTHLGREQSRRDDLEALGHVFLYFLRGGLPWQGLKAATN 227
VdCK1      KQHIPYRERKSLSGTARYMSINTHLGREQSRRDDLEALGHVFMYFLRGGLPWQGLKAATN 227
           ********:*************************:**:****:***** ******** .*

YCk1       KQKYEKIGEKKRSTNVYDLAQGLPVQFGRYLEIVRSLSFEECPDYEGYRKLLLSVLDDLG 343
YCk2       KQKYEKIGEKKRLTNVYDLAQGLPIQFGRYLEIVRNLSFEETPDYEGYRMLLLSVLDDLG 350
CnCk1      KQKYEKIGEKKQTTPIAELVEGYPNEFSIYLNYVRKLTFDETPDYDFLRGLFDLALSNSG 308
MoCk1      KQKYEKIGEKKQTTAIKELCEGFPIQFEKYLTYVRNLGFEDTPDYDYLRELFTQALKESG 287
FoCk1      KQKYEKIGEKKQTTAIKDLCEGFPEEFSKYLTYVRNLGFEDTPDYDYLRELFTQALKNTG 287
VdCK1      KQKYEKIGEKKQTTQIRDLCEGFPDELNKYLTYVRNLGFEDTPDYDYLRELFTQALKNTG 287
           ***********: * : :* :* * ::  **  **.* *:: ***:  * *:  .*.: *

YCk1       ETADGQYDWMKLND--GRGWDLNINKKPNLHGY--GHPNPPNEKSRKHRNKQLQMQQLQM 399
YCk2       ETADGQYDWMKLNG--GRGWDLSINKKPNLHGY--GHPNPPNEKSKRHRSKNHQYSSPDH 406
CnCk1      EMDDGVYDWMMLNN--GQGWEASGRQSAAQAE------------IARHN-------TRGR 347
MoCk1      GVEDGEYDWMKVSKDKKNDWDRQ------AMHNPSQRPGASNMELHGAS-------RGAH 334
FoCk1      EVEDGEYDWMKISKDSGKGWDS-KSHSGAYLHNPNVRPGPSQMELHSGH-------RPGN 339
VdCK1      EVEDGEYDWMKISKDSGKGWDGSKGHNNAYLHNPNARPGPSQMELHSGQ-------RGGA 340
              ** **** :.    ..*:

YCk1       QQ-----------------------------LQQQQQQQQYA-QKTEADMRNSQYKPKL 428
YCk2       HHHYNQQQQQQQAQAQAQAQAQAKVQQQQLQQAQAQQQANRYQLQPDDSHYDEEREASKL 466
CnCk1      DREYRDRVDKLR--------------NG----SAAN--------QPSPGKPRK---SGSA 378
MoCk1      ASDLRDPRARLT--------------VDR---LNAA--------QPPPPSPIKAN-QGKD 368
FoCk1      TTS-HQQAQNLT--------------VGR---LNAA--------QPPPPSPIKQMGKQRD 373
VdCK1      SAA-HAQAQNLT--------------VSR---LNAA--------QPPPPSPIKQMNK--G 372
                                                       *       :

YCk1       DPTSYEAYQHQTQQKYLQEQQKRQQQQKLQEQQLQEQQLQQQQQQQQQLRATGQPPSQPQ 488
YCk2       DPTSYEAYQQQTQQKYAQQQQKQMQQKSKQF---------------ANTGANGQTN---- 507
CnCk1      LPNVSN----QAM--------------VGVSA--------------PSPLPQSRRQSQ 404
MoCk1      RRNAPGAIMASRG---------------T-AGG---------------LRDMATPTGSTQ 397
FoCk1      RPSAPGALSAQRG---------------SGVGG---------------LRDMATPTGSTQ 403
VdCK1      RPNAPGALGAQRS---------------SGVGG---------------LRDMATPAGSTQ 402
             .       .

YCk1       AQTQSQQFGARYQPQQQPSA-----ALRTPEQHPNDDNSSLAASHKGFFQKLGCC----   538
YCk2       ----------KYPYNAQPTA-----NDEQN-AKNAAQDRNSNKSSKGFFSKLGCC----   546
CnCk1      QAVQSQ---AGLQPISPMNVNSRGGNTAANSQ-QLGEGDYHQENKGNGFIRFITCGCFR   459
MoCk1      AQFQNS---TQNLPQRPMH---QQSNMTQPQL-NNGRPAEPEPTGFQKFMKVLCCG---   446
FoCk1      AQFQNS---AQNLPQQPRT--SQQGQATQPAQ-ASGQQANPQPSGFQKLMKTLCCG---   453
VdCK1      AQFQNS---NRNLPQTAP-----QQSPAQPAQ-PSGRSPEPQPSGMQKFMKVLCC----   448
                                                      .    : :   *
```

Supplementary Figure S1. Amino acid sequence alignment of the *Yck1* (YHR135C) *and Yck2* (YNL154C) proteins from *S. cerevisiae* with Ck1 proteins from *C. neoformans* (CNA05390), *M. oryzae* (MGG_08097), *F.oxysporum* Ck1 (FOXG_05428) and *V. dahliae* (VDAG_08537).

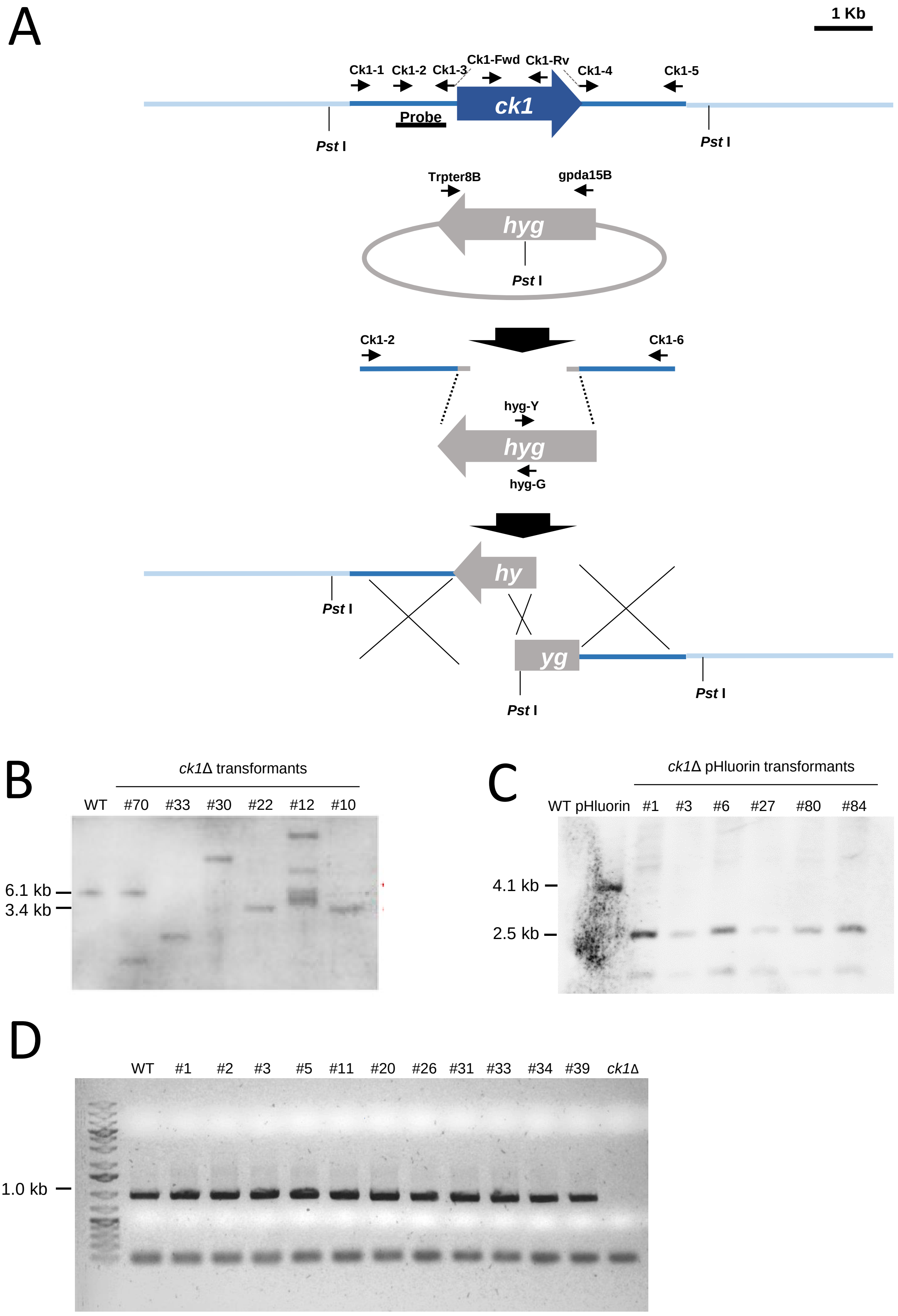

Supplementary Figure S2. Targeted replacement of the *ck1* gene in the *F. oxysporum* wild type and pHluorin-expressing strain (**A**) Physical maps of the *F. oxysporum ck1* locus and of the split-marker gene replacement constructs obtained by fusion PCR. Relative positions of restriction sites and PCR primers are indicated. *hyg*, hygromycin resistance gene. (**B**) Southern blot analysis of genomic DNA of the wild type strain (WT) and six independent transformants, treated with *Pst* I, separated on a 0.7% agarose gel, transferred to a nylon membrane and hybridized with the DNA probe indicated in (A). (**C**) Southern blot analysis of genomic DNA of the pHluorin-expressing background strain (pHluorin) and six independent transformants, treated with *Nsi*I, separated on a 0.7% agarose gel, transferred to a nylon membrane and hybridized with the DNA probe indicated in (A). (**D**) PCR amplification of genomic DNA of the WT, the *ck1*Δ and eleven independent complemented transformants, using primers Ck1-Fwd and Ck1-Rv indicated in (A).

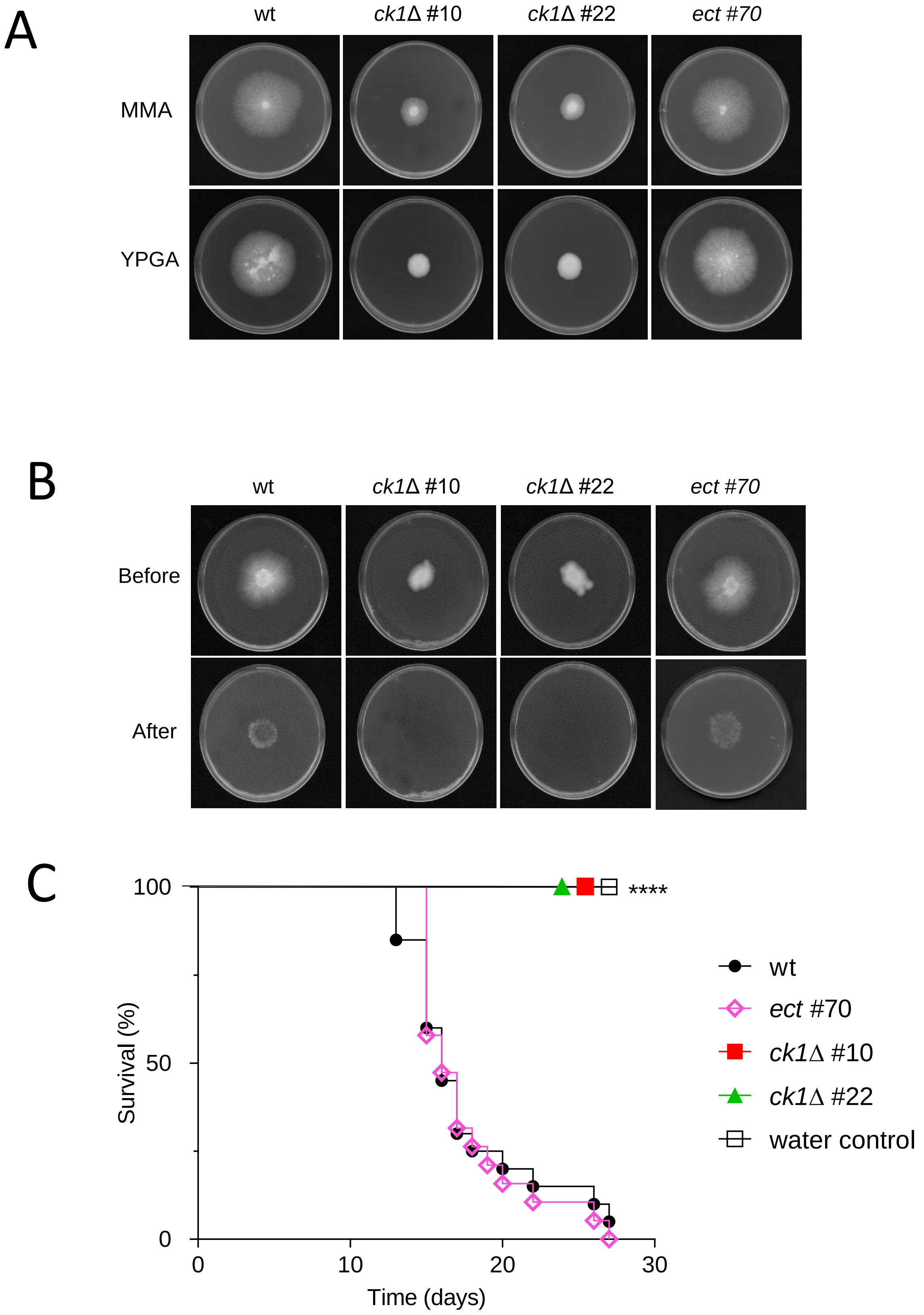

Supplementary Figure S3. Ck1 is required for growth, invasion and pathogenicity of *F. oxysporum*. (**A**) Aliquots of 5x10^4 fresh microconidia of the indicated strains were spot-inoculated on MMA or YPGA plates. Colonies were imaged 3 days after inoculation. (**B**) Aliquots of 5x10^4 fresh microconidia of the indicated strains were spot-inoculated on the top cellophane membranes placed on unbuffered MMA plates. Colonies were imaged 3 days after inoculation (before), then the cellophane membrane with the fungal colony was removed and plates were incubated for an additional day to visualize the presence on the plate, indicative of penetration through the cellophane (after). (**C**) Kaplan-Meier plots showing survival of tomato plants (cv. Moneymaker) inoculated by dipping roots into a suspension of 5x10^6 fresh microconidia/mL of the indicated fungal strains. Survival of tomato plants was recorded for 27 days. Twenty plants were used per treatment. $p<0.0001$(****) versus wt according to log-rank test. All data shown are from one representative experiment. Experiments were performed twice with similar results.

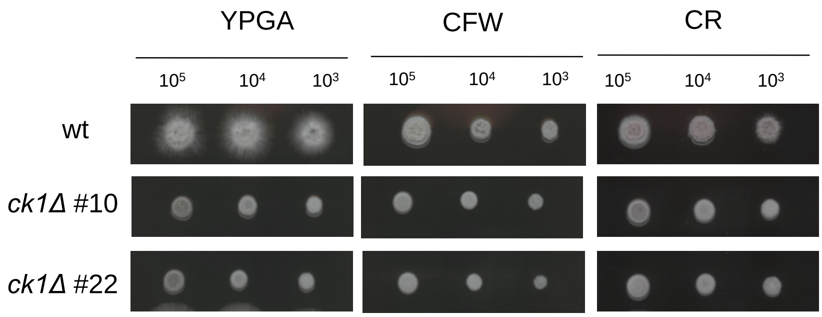

Supplementary Figure S4. Ck1 is not required for the response of *F. oxysporum* to cell wall stress. Aliquots of 10^5, 10^4 and 10^3 fresh microconidia of the indicated strains were spot-inoculated on YPGA plates in the absence or presence of 50µg/ml Calcofluor White (CFW) or 100µg/ml Congo Red (CR). The plates were imaged after two days of incubation at 28°C. Images are from one representative experiment. Experiments were performed twice, each with three independent plates per growth condition.

```
S.cerevisiae           MTDTSSSSSSSSASSV--S-------AHQPTQEKPAK--TYDDAASESSDDDDIDALIE-     48
V.dahliae              MA--ESNSAAAPALNTPIESHRFEEKDRPLDTGVDAP--VKPKVEEEEDEDEDIDALIE-     55
M.oryzae               MADSTQPTGAGPAINTPIESGKFDEKEGLAHNPAAKP--K---VADEEEEDEDIDALIE-     54
N.crassa               MA--DHSASGAPALSTNIESGKFDEKAAEAAAYQPKP--K-----VEDDEDEDIDALIE-     50
T.reesei               MA--DDKAVGAPALDTNIESGNFDEKRAQAPAGVPP---K-KAPAEEEEEDEDIDALIE-     53
F.graminearum          MA--EEKAVGAPALDTNIETGGFDEKRGQAP-ATHNP--K-APVAEDEEPDEDMDALIE-     53
F.oxysporum            MA--EEKAAGAPALDTNIETGGFDEKRGQAPPPTHAP--K-APVAEDEEPDEDMDALIE-     54
F.verticillioides      MA--EEKATGAPALDTNIETGGFDEKRGQAAPPTHAP--K-APVAEDEEPDEDMDALIE-     54
C.neoformans           MGLTNR---------------KNHKKDPEAGDPETEAKRQ-EEDKKKKYSGEEYDVLLKY     44
A.nidulans             MAERKI---------------SYA-ADVENGDHS------RPTDVNDSAGLDEYGALNRY     38
Z.triciti              MAAPRV---------------SFSDKDLENGEGGPERSRKWSQAPGNIEDLDEYTALQKY     45
                       *                                             .    ::  .* .

S.cerevisiae           --ELQSNH--GVDD-----------------EDSDNDGPVAAGEARPVPEEYLQTDPSYG     87
V.dahliae              --DLESQD--GHIE-----------------E-DE-EEETAVGGARVIPEDQLATDTRLG     92
M.oryzae               --DLESVD--GHGD-----------------LEEE-EEEAGPGAARVIPEDLLQTSTRTG     92
N.crassa               --DLESHD--GHD------------------AEEE-EEEATPGGGRVVPEDMLQTDTRVG     87
T.reesei               --DLESED--GHA------------------FDDE-EEETNPATGRVVPEDMLQTDSRLG     90
F.graminearum          --DLESED--GHE------------------IDDD-E-EATPGGGRVVPEDQLQTDSRVG     89
F.oxysporum            --DLESED--GHA------------------FDEE-E-ETQPGGGRVVPEDQLQTDSRVG     90
F.verticillioides      --DLESED--GHA------------------FDDE-E-ETQPGGGRVVPEDQLQTDSRVG     90
C.neoformans           VADQQEKIKKGGGDDEKEDEENVKYIRKWYTPWKKTKV---ETGGKKVPPDWLGTDRQKG     101
A.nidulans             ISTARDNRR-GSTSSA-GALSMKQKKKPWYKFWAK-AGGENGEEGFVAPEDWLETDL-NG     94
Z.triciti              ISTYRDPKL-AQQDEVANAHAESQKKKPWQ-FWKKAPKAEDDGDAMVVPEDWLNADIRQG     103
                           ..    .                       .         .   * : * :.   *

S.cerevisiae           LTSDEVLKRRKKYGLNQMADEKESLVVKFVMFFVGPIQFVMEAAAILAAGLSDWVDFGVI     147
V.dahliae              LTEAEVLNRRRKYGANQMKEEKENLILKFFVME--------SRCPSSAAGLEDWVDFGVI     144
M.oryzae               LTEQEVQARRRKYGLNQMKEEKENLIMKFLGYFIGPVQFVMEAAVILAAGLQHWVDFAVI     152
N.crassa               LTSEEVVQRRRKYGLNQMKEEKENHFLKFLGFFVGPIQFVMEGAAVLAAGLEDWVDFGVI     147
T.reesei               LTEAEVLARRRKYGLNQMKEEKENLVLKFLGFFIGPIQFVMEAAAVLAAGLQDWVDFGVI     150
F.graminearum          LTEAEVIARRKKWGLNAMKEEQENMILKFLMFFVGPIQFVMEAAAVLAAGLEDWIDFGVI     149
F.oxysporum            LTEAEVINRRRKWGLNQMKEERENMILKFLMFFVGPIQFVMEAAAVLAAGLEDWIDFGVI     150
F.verticillioides      LTEAEVINRRRKWGLNQMKEERENMILKFLMFFVGPIQFVMEAAAVLAAGLEDWIDFGVI     150
C.neoformans           LSSSEIEERRKHSGWNELESPNENQFLKFISYFRGPILYVMELAVILAAGLRDWIDFGVI     161
A.nidulans             LPSSQIEPRRKRGGWNELTTEKTNFFVQFIGYFRGPILYVMELAVLLAAGLRDWIDLGVI     154
Z.triciti              ITNADVESRRKKFGWNEISTDKENLFIKFLTFFTGPILYVMELAVLLAAGLRSWIDFGVI     163
                       : . ::  **:: * * :   . . .::*.           . .   ****  *:*:.**

S.cerevisiae           CGLLMLNAGVGFVQEFQAGSIVDELKKTLANTAVVIRDGQLVEIPANEVVPGDILQLEDG     207
V.dahliae              IALLLLNAVVGFYQEFQAGSIVDELKKTLALKAVVLRDGQLKEVEAHEVVPGDILQVEDG     204
M.oryzae               CALLLLNACVGFIQEFQAGSIVDELKKTLALKAVVLRDGQLKEVEAPEVVPGDILQVEEG     212
N.crassa               CGLLLLNAVVGFVQEFQAGSIVDELKKTLALKAVVLRDGTLKEIEAPEVVPGDILQVEEG     207
T.reesei               CGLLMLNACVGFIQEFQAGSIVEELKKTLALKAVVLRDGTLKEIEAPEVVPGDILQVEEG     210
F.graminearum          CALLLLNACVGFIQEYQAGSIVEELKKTLALKAVVLRDGTLKEIEAPEVVPGDILQVEEG     209
F.oxysporum            CALLLLNACVGFIQEFQAGSIVEELKKTLALKAVVLRDGTLKEVEAPEVVPGDILQVEEG     210
F.verticillioides      CALLLLNACVGFIQEFQAGSIVEELKKTLALKAVVLRDGTLKEVEAPEVVPGDILQVEEG     210
C.neoformans           IGILFLNAGVGWYQEKQAGDIVAQLKAGIALKADVIRDGKEQEIEARELVPGDILVLEEG     221
A.nidulans             IGILMLNAVVGWYQEKQAADVVASLKGDIAMKAVVKRDGQEQEILARELVTGDIVVIEEG     214
Z.triciti              IAILLLNAAVGWYQEKQAADVVASLKGDIAMKATVVRDGQEQDIKARELVPGDIVVIEEG     223
                        .:*:*** **: ** **..:* .**  :* .* * ***   :: * *:* ***: :*:*

S.cerevisiae           TVIPTDGRIVTED-------------------------------------------CFL     223
V.dahliae              TIIPADGRIVTDD-------------------------------------------AFL     220
M.oryzae               TIIPADGRIVTDD-------------------------------------------AYL     228
N.crassa               TIIPADGRIVTDD-------------------------------------------AFL     223
T.reesei               TIIPADGRIVTEG-------------------------------------------AFL     226
F.graminearum          TIIPADGRFVTEG-------------------------------------------CFC     225
F.oxysporum            TIIPADGRFVTEG-------------------------------------------CFV     226
F.verticillioides      TIIPADGRFVTEG-------------------------------------------CFV     226
C.neoformans           KTIAADAKIIGDYEDKDGSKSKDIL-DRV--EKSKHSKGGDDDD----EDDGPDKGPSLC     274
A.nidulans             TIVPADVRLICDYDKPETYETYKEYLATANDDTLKE-----NDDDDDDHGIEARLGVSLV     269
Z.triciti              QSVPADSRLICDYEHPEDFEKYKELREQHALNPEEDPAGSEDAEGEEGEG-IQHQGHSII     282
                         : :* ::: :                                             .
```

```
S.cerevisiae        QIDQSAITGESLAVDKHYGDQTFSSSTVKRGEGFMVVTATGDNTFVGRAAALVNKAAGGQ    283
V.dahliae           QVDQSAITGESLAVDKHKGDNCYASSAVKRGEAFIVITATGDSTFVGRAAALVNAASAGT    280
M.oryzae            QVDQSAITGESLAVDKHKGDQCYASSAVKRGEAFVVITATGDSTFVGRAAALVNAASAGT    288
N.crassa            QVDQSALTGESLAVDKHKGDQVFASSAVKRGEAFVVITATGDNTFVGRAAALVNAASGGS    283
T.reesei            QVDQSAITGESLAVDKHKGDSCYASSAVKRGEAFLVVTATGDNTFVGRAAALVSQSSGGT    286
F.graminearum       QVDQSAITGESLAVDKHHGDNCYASSAVKRGEAFVIVTATGDNTFVGRAAALVSQSAGGT    285
F.oxysporum         QVDQSAITGESLAVDKHAGDNCYASSAVKRGEAFVIVTATGDNTFVGRAAALVSQSAGGT    286
F.verticillioides   QVDQSAITGESLAVDKHAGDNCYASSAVKRGEAFIIVTATGDNTFVGRAAALVSQSAGGT    286
C.neoformans        SVDQSAITGESLAVDKFIGDVAYYTCGVKRGKCFGVVTVSAKGSFVGRTASLVSS-SNEK    333
A.nidulans          AVDQSAITGESLAVDKYMADTCYYTTGCKRGKAYAIVTATAKHSFVGKTAALVQG-AQDQ    328
Z.triciti           AADQSSITGESLAVDKYMGDTVYYTTGCKRGKAYGVVLTSAKFSFIGRTATLVQG-AKDQ    341
                      ***::*********. .*  : :   ***: : :: .:.. :*:*::*:**.  :

S.cerevisiae        GHFTEVLNGIGIILLVLVIATLLLVWTACFYRTNGIV-------RILRYTLGITIIGVPV    336
V.dahliae           GHFTEVLNGIGTVLLILVVATLLIVWVSGFYRSNDIV-------EILRFTLAILIVGVPV    333
M.oryzae            GHFTEVLNGIGTVLLILVVFTNLVVWISSFYRNNSIV-------QILEFTLAITIIGVPV    341
N.crassa            GHFTEVLNGIGTILLILVIFTLLIVWVSSFYRSNPIV-------QILEFTLAITIIGVPV    336
T.reesei            GHFTEVLNGIGTILLVLVVLTLLVVWISSFYRSNGIV-------DILRFTLAITIVGVPV    339
F.graminearum       GHFTEVLNGIGTILLVLVVATLLIVWVSSFYRSNGIV-------DILRFTLAITIVGVPV    338
F.oxysporum         GHFTEVLNGIGTILLVLVVATLLIVWVSSFYRSNGIV-------DILRFTLAITIVGVPV    339
F.verticillioides   GHFTEVLNGIGTILLVLVVATLLVVWVSSFYRSNGIV-------DILRFTLAITIVGVPV    339
C.neoformans        GHFQIVLGGIGTTLLVMVIAFIFAVWIGGFFRGTGIATPR--ENNLLVYALIFFIIGVPV    391
A.nidulans          GHFKAVMDNIGTSLLVLVMFWILAAWIGGFYRHLKIATPEHSDNTLLHWTLILLIIGVPV    388
Z.triciti           GHFKAIMNSIGTALLVLVMFWILVAWIGGFFRGINLATPEASSNTLLHYVLILFIIGVPV    401
                    ***   ::..**  **::*:   : .* . *:*   :.         :* :.* : *:****

S.cerevisiae        GLPAVVTTTMAVGAAYLAKKQAIVQKLSAIESLAGVEILCSDKTGTLTKNKLSLHEPYTV    396
V.dahliae           GLPAVVTTTMAVGAAYLAKKKAIVQKLSAIESLAGVEILCSDKTGTLTKNKLSLAEPYTV    393
M.oryzae            GLPAVVTTTMAVGAAYLAKKKAIVQKLSAIESLAGVEILCSDKTGTLTKNKLSLSEPYTV    401
N.crassa            GLPAVVTTTMAVGAAYLAKKKAIVQKLSAIESLAGVEILCSDKTGTLTKNKLSLHDPYTV    396
T.reesei            GLPAVVTTTMAVGAAYLAKKQAIVQKLSAIESLAGVEILCSDKTGTLTKNKLSLSEPYTV    399
F.graminearum       GLPAVVTTTMAVGAAYLAKKQAIVQKLSAIESLAGVEILCSDKTGTLTKNKLSLAEPFCV    398
F.oxysporum         GLPAVVTTTMAVGAAYLAKKQAIVQKLSAIESLAGVEILCSDKTGTLTKNKLSLAEPFCV    399
F.verticillioides   GLPAVVTTTMAVGAAYLAKKQAIVQKLSAIESLAGVEILCSDKTGTLTKNKLSLAEPFCV    399
C.neoformans        GLPVVTTTTLAVGAAYLAKRKAIVQKLTAIESLAGVDILCSDKTGTLTANKLSLNEPYIA    451
A.nidulans          GLPVVTTTTLAVGAAYLAEQKAIVQKLTAIESLAGVDILCSDKTGTLTANQLSIREPYVN    448
Z.triciti           GLPVVTTTTLAVGAAYLAKQQAIVQKLTAIESLAGVDVLCSDKTGTLTANQLSIREPYVA    461
                    ***.*.***:********:::******:********::********** *:**: :*:

S.cerevisiae        EGVSPDDLMLTACLAASRKKKGLDAIDKAFLKSLKQYPKAKDALT-KYKVLEFHPFDPVS    455
V.dahliae           AGVEPDDLMLTACLAASRKKKGIDAIDKAFLKSLRYYPRAKSVLS-KYSVLEFFPFDPVS    452
M.oryzae            AGVEPEDLMLTACLAASRKKKGMDAIDKAFLKSLKYYPRAKAALS-KYKVLEFHPFDPVS    460
N.crassa            AGVDPEDLMLTACLAASRKKKGIDAIDKAFLKSLKYYPRAKSVLS-KYKVLQFHPFDPVS    455
T.reesei            QGVDPDDLMLTACLAASRKKKGIDAIDKAFLKSLKFYPRAKSVLS-KYKVIEFHPFDPVS    458
F.graminearum       AGVEPDDLMLTACLAASRKKKGIDAIDKAFLKALKFYPRAKGVLS-KYKVLDFHPFDPVS    457
F.oxysporum         AGVEPDDLMLTACLAASRKKKGIDAIDKAFLKALKYYPRAKSVLS-KYKVLDFHPFDPVS    458
F.verticillioides   AGVEPDDLMLTACLAASRKKKGIDAIDKAFLKALKYYPRAKSVLS-KYKVLDFHPFDPVS    458
C.neoformans        PDVDPNWFMAVAVLASSHNVLGLDPIDKVTIVGLKDYPKAQEMLKGGWKTHKFTPFDPVS    511
A.nidulans          EGVDVNWMMAVAAIASNHNVKNLDPIDKVTILTLRRYPKAREILARNWVTEKYTPFDPVS    508
Z.triciti           EGEDVNWMMACAALASSHNIKSLDPIDKVTILTLKRYPKARDILKDDWKTEKFTPFDPVS    521
                     . . : :*  * :*:.::  .:* ***. :  *: **:*:  *   : . .: ******

S.cerevisiae        KKVTAVVESPEGERIVCVKGAPLFVLKTVEEDHPIPEDVHENYENKVAELASRGFRALGV    515
V.dahliae           KKVTAVVQSPQGERITCVKGAPLFVLKTVEEDHPLDPEIDMAYKNKVAEFATRGFRSLGV    512
M.oryzae            KKVTAIVESPQGEIITCVKGAPLFVLKTVEEDHEIPEAVDQDYKNKVAEFATRGFRSLGV    520
N.crassa            KKVVAVVESPQGERITCVKGAPLFVLKTVEEDHPIPEEVDQAYKNKVAEFATRGFRSLGV    515
T.reesei            KKVTAVVESPQGERITCVKGAPLFVLKTVEEDHPIPEEIDKAYKNQVAEFATRGFRSLGV    518
F.graminearum       KKVQAVVESPQGERIICVKGAPLFVLKTVEEDHPIPEEVDSAYKNQVAEFATRGFRSLGV    517
F.oxysporum         KKVQAVVESPQGERIICVKGAPLFVLKTVEEDHPIPEEVDAAYKNQVAEFATRGFRSLGV    518
F.verticillioides   KKVQAVVESPQGERIICVKGAPLFVLKTVEEDHPIPEEIDAAYKNTVAEFATRGFRSLGV    518
C.neoformans        KRITAEVE-KEGKHYTCAKGAPNAILKLAKF----APDTVSAYRAQSQQFASRGFRSLGV    566
A.nidulans          KRITTICT-CDGVRYTCAKGAPKAILAMSEC----SPEEAQKFREKASEFARRGFRSLGV    563
Z.triciti           KRITTICT-LRGDRFTCAKGAPKAVLNLTEC----SKETADMFKDKATEFARRGFRSLGV    576
                    *:: :      *    *.****  :*    :             :.    ::* ****:***

S.cerevisiae        ARKRGEGHWEILGVMPCMDPPRDDTAQTVSEARHLGLRVKMLTGDAVGIAKETCRQLGLG    575
V.dahliae           ARKRGEGNWEILGIMPCSDPPRHDTARTVNEAKNLGLSVKMLTGDAVGIARETSRQLGLG    572
M.oryzae            ARKRGEGAWEILGIMPCSDPPRHDTARTINEAKNLGLSIKMLTGDAVGIARETSRQLGLG    580
N.crassa            ARKRGEGSWEILGIMPCMDPPRHDTYKTVCEAKTLGLSIKMLTGDAVGIARETSRQLGLG    575
```

```
T.reesei            ARKRGEGAWEILGIMPCSDPPRHDTARTINEAKQLGLSIKMLTGDAVGIARETSRQLGLG    578
F.graminearum       ARKRGEGAWEILGIMPCSDPPRHDTARTINEAKRLGLSIKMLTGDAVGIARETSRQLGLG    577
F.oxysporum         ARKRGEGAWEILGIMPCSDPPRHDTARTINEAKRLGLSIKMLTGDAVGIARETSRQLGLG    578
F.verticillioides   ARKRGEGAWEILGIMPCSDPPRHDTARTINEAKRLGLSIKMLTGDAVGIARETSRQLGLG    578
C.neoformans        AVKEEGKDWELLGMLCMFDPPRVDTAKTIGEAHDLGIQVKMLTGDAVAIAKETCKQLGLK    626
A.nidulans          AVQKEGEPWQLLGMYPMFDPPREDTAHTIAEAQHLGLSVKMLTGDALAIAKETCKMLALS    623
Z.triciti           AYQKNNDPWVLLGMLSMFDPPREDTAQTIIEAQQLGVPVKMLTGDAIAIAKETCKMLALG    636
                    * :.    * :**:    **** **  :*: **: **:  :*******:.**:**.: *.*

S.cerevisiae        TNIYNAERLGLGGGGDMPGSELADFVENADGFAEVFPQHKYRVVEILQNRGYLVAMTGDG    635
V.dahliae           TNIFNADRLGLGGGGDMPGSEVYDFVEAADGFAEVFPQHKYNVVEILQQRGYLVAMTGDG    632
M.oryzae            TNVYNAERLGLGGGGDMPGSEVYDFVEAADGFAEVFPQHKYNVVEILQQRGYLVAMTGDG    640
N.crassa            TNIYNAERLGLGGGGDMPGSEVYDFVEAADGFAEVFPQHKYNVVEILQQRGYLVAMTGDG    635
T.reesei            TNVYNAERLGLGGGGDMPGSEVYDFVEAADGFAEVFPQHKYSVVEILQQRGYLVAMTGDG    638
F.graminearum       TNVYNAERLGLGGGGDMPGSEVYDFVEAADGFAEVFPQHKYNVVEILQQRGYLVAMTGDG    637
F.oxysporum         TNVYNAERLGLGGGGDMPGSEVYDFVEAADGFAEVFPQHKYNVVEILQQRGYLVAMTGDG    638
F.verticillioides   TNVYNAERLGLGGGGDMPGSEVYDFVEAADGFAEVFPQHKYNVVEILQQRGYLVAMTGDG    638
C.neoformans        TNVYDSEKLIG---GGMAGSDIRDFVEAADGFAEVFPEHKYQVVNLLQERGHLTAMTGDG    683
A.nidulans          TKVYDSERLIH---GGLAGSAQHDLVEKADGFAEVFPEHKYQVVEMLQQRGHLTAMTGDG    680
Z.triciti           TKVYNSQKLIH---GGLSGTTQHDLVERADGFAEVFPEHKYQVVEMLQQRGHLTAMTGDG    693
                    *:::::::*     *.: *:   *:** *********:*** **::**:**:*.******

S.cerevisiae        VNDAPSLKKADTGIAVEGATDAARSAADIVFLAPGLSAIIDALKTSRQIFHRMYSYVVYR    695
V.dahliae           VNDAPSLKKADTGIGMFDVPRSST------------------------------VSILP    661
M.oryzae            VNDAPSLKKADTGIAVEGASDAARSAADIVFLAPGLGAIIDALKTSRQIFHRMYAYVVYR    700
N.crassa            VNDAPSLKKADTGIAVEGSSDAARSAADIVFLAPGLGAIIDALKTSRQIFHRMYAYVVYR    695
T.reesei            VNDAPSLKKADTGIAVEGASDAARSAADIVFLAPGLGAIIDALKTSRQIFHRMYAYVVYR    698
F.graminearum       VNDAPSLKKADTGIAVEGASDAARSASDIVFLAPGLGAIIDALKTSRQIFHRMYAYVVYR    697
F.oxysporum         VNDAPSLKKADTGIAVEGASDAARSASDIVFLAPGLGAIIDALKTSRQIFHRMYAYVVYR    698
F.verticillioides   VNDAPSLKKADTGIAVEGASDAARSAADIVFLAPGLGAIIDALKTSRQIFHRMYAYVVYR    698
C.neoformans        VNDAPSLKKADCGIAVEGASDAARTAADVVFLDEGLSTIITAIKVARQIFHRMKAYIIYR    743
A.nidulans          VNDAPSLKKADCGIAVEGSTEAAQAAADIVFLAPGLSTIVDAIKLARQIFQRMKAYIQYR    740
Z.triciti           VNDAPSLKKADCGIAVEGSSEAAQAAADIVFLAPGLSTIVFAIKTARQIFQRMKAYIQYR    753
                    *********** **.:  .   ::

S.cerevisiae        IALSLHLEIFLGLWIAILDNSLDIDLIVFIAIFADVATLAIAYDNAPYSPKPVKWNLPRL    755
V.dahliae           LTRSQLSKLYLGLWIAILNRSLNIELVVFIAIFADIATLAIAYDNAPYSKAPVKWNLPKL    721
M.oryzae            IALSIHLEIYLGILIAALNQSLNINLVAFIAIFADIATLAIAYDNAPYSKSPVKWNLPKL    760
N.crassa            IALSIHLEIFLGLWIAILNRSLNIELVVFIAIFADVATLAIAYDNAPYSQTPVKWNLPKL    755
T.reesei            IALSLHMEIFLGLWIAILNRSLNIELVVFIAIFADIATLAIAYDNAPYSQTPVKWNLPKL    758
F.graminearum       IALSLHMEIFLGLWIAILNRSLNIELVVFIAIFADIATLAIAYDNAPFSQTPVKWNLPKL    757
F.oxysporum         IALSLHMEIFLGLWIAILNRSLNIELVVFIAIFADIATLAIAYDNAPFSQTPVKWNLPKL    758
F.verticillioides   IALSLHMEFFLGLWIAILNKSLNIELVVFIAIFADIATLAIAYDNAPFSQTPVKWNLPKL    758
C.neoformans        IALCVHLEVYLMLSILILNETIRVDLVVFLAIFADVATIAIAYDRAPYAHQPVEWQLPKV    803
A.nidulans          IALCIHLELYLVTSMIIINETIKADLIVFIALFADLATIAVAYDNAHFEARPVEWQLPKI    800
Z.triciti           IALCLHLEIYLVTSIIIIREVIASELIVFIALFADLATVAIAYDNAHSEQRPVEWQLPKI    813
                    :: .   :.:*   :  :  .  :  :*:.*:*:***:**:*:***.*     **:*:**::

S.cerevisiae        WGMSIILGIVLAIGSWITLTTMFL--PKGGIIQNFGAMNGIMFLQISLTENWLIFITRAA    813
V.dahliae           WGISVILGIVLAIGTWITVTTMYAHGPNGGIVQNFGNLDEVVFLQVSLTENWLIFITRAN    781
M.oryzae            WGMSVLLGVVLAIGTFITITTMFVHGEDGGIVQNNGQIDAVVFLEISLTENWLIFITRAN    820
N.crassa            WGMSVLLGVVLAVGTWITVTTMYAQGENGGIVQNFGNMDEVLFLQISLTENWLIFITRAN    815
T.reesei            WGMSVLLGTVLAIGTWIALTTMYAGGQNGGIVQNFGNIDEVVFLEISLTENWLIFITRAN    818
F.graminearum       WGMSVLLGVVLAVGTWIALTTMLANSEDGGIVQNFGKIDEVLFLEISLTENWLIFITRAN    817
F.oxysporum         WGMSVLLGVVLAVGTWIALTTMYANSEDGGIVQNFGKIDEVLFLEISLTENWLIFITRAN    818
F.verticillioides   WGMSVLLGVVLAVGTWIALTTMYANSEDGGIVQNFGKIDEVLFLEISLTENWLIFITRAN    818
C.neoformans        WIISTIMGLLLAAGTWIIRATLWI--DNGGIVQNFGSTQEILFLEVALTESWVIFITRLA    861
A.nidulans          WVISVVLGVLLAAGTWIMRASLFL--ENGGIIQNFGSPQPMLFLEVSLTENWLIFVTRGG    858
Z.triciti           WIISVILGIELAIATWIARGTFFM--PGGGLIQNYGNFEEILFLEIALTENWLIFITRGA    871
                    * :* ::*  ** .::*   ::      **::** *   : ::**:::***.*:**:**

S.cerevisiae        GP--FWSSIPSWQLAGAVFAVDIIATMFTLFGWWSE-----------------NW-TDI    852
V.dahliae           GP--FWSSIPSWQLSGAIFIVDILATLFCIFGWFEH-----------------GQ-TSI    820
M.oryzae            GP--FWSSIPSWQLTGAIFVVDIIASCFAIWGWFVG-----------------NKMTHI    860
N.crassa            GP--FWSSIPSWQLSGAIFLVDILATCFTIWGWFEH-----------------SD-TSI    854
T.reesei            GP--FWSSIPSWQLSGAILVVDIIATLFCVFGWFIG-----------------ED-TSI    857
F.graminearum       GP--FWSSIPSWQLSGAILIVDILATLFCIFGWFVG-----------------GQ-TSI    856
F.oxysporum         GP--FWSSIPSWQLSGAILIVDILATLFCIFGWFVG-----------------GQ-TSI    857
F.verticillioides   GP--FWSSIPSWQLSGAILIVDILATLFCIFGWFVG-----------------GQ-TSI    857
```

```
C.neoformans          QEPGTPNVWPSFQLVAAVIGVDALATIFALFGWISGDAP-------------HGGWTDV     907
A.nidulans            ------KTWPSWQLVGAIFVVDVLATLFCVFGWLAGDYVETSPPS---QATFSTNNDTDI    909
Z.triciti             ------QTLPSWQLVGAILGVDILATLFCIFGWLNSSIYQRPLPSPMSTFQQTANGHTDV    925
                            .  **:** .*:: ** :*: * ::**                    * :

S.cerevisiae          VTVVRVWIWSIGIFCVLGGFYYEMSTSEAFDRLMNGKPMKEKKSTRSVEDFMAAMQRVST    912
V.dahliae             VAVVRIWIFSFGVFCVCAGVYYILQDNAGFDNMMHGKSPKGSQKQRSLEDFVVSLQRVST    880
M.oryzae              VAVVRIWVFSFGVFCIMGGLYYILQDSQGFDNLMHGKSPKGNQKQRSLEDFVVSLQRVST    920
N.crassa              VAVVRIWIFSFGIFCIMGGVYYILQDSVGFDNLMHGKSPKGNQKQRSLEDFVVSLQRVST    914
T.reesei              VAVVRIWIFSFGIFAIMGGLYYFLQGSTGFDNLMHGKSPKQNQKQRSLEDFVVSLQRVST    917
F.graminearum         VAVVRIWIFSFGVFCVMGGLYYFMQGSTGFDNLMHGKSPKQNQKQRSLEDFVVSLQRVST    916
F.oxysporum           VAVVRIWIFSFGVFCVMGGLYYFMQGSTGFDNLMHGKSPKQNQKQRSLEDFVVSLQRVST    917
F.verticillioides     VAVVRIWIFSFGVFCVMGGLYYFMQGSTGFDNLMHGKSPKQNQKQRSLEDFVVSLQRVST    917
C.neoformans          VTVVKIWCFSFGVVIIILLVYLMLNSIRWLDHIGRKSR---SKKNEKLENFLTDLQRLTI    964
A.nidulans            VTVVVIWAYSIGVTIIIAVVYYLLTIIPALDNLGRKNR---SVVDTKVENLLNHLSKLAI    966
Z.triciti             VTVVVVWMFSIGVMIVIAITYYLLNKIPGLADLGRQNR---SLHDTQMENIIGHLSKLAL    982
                      *:** :* :*:*:  :    *  :     :  : . .    .    .:*:::  :.:::

S.cerevisiae          QHEKET*-----------------------------------------------------    918
V.dahliae             QHEKSQ------------------------------------------------------    886
M.oryzae              QHEKSQ------------------------------------------------------    926
N.crassa              QHEKSQ------------------------------------------------------    920
T.reesei              QHEKSQ------------------------------------------------------    923
F.graminearum         QHEKSQ------------------------------------------------------    922
F.oxysporum           QHEKSQ------------------------------------------------------    923
F.verticillioides     QHEKSQ------------------------------------------------------    923
C.neoformans          VHETDHNGSYY-RFASKKEEEESGDNGKKDDKKDEAKSADTKKQESNAKKGDDEKKKDDD    1023
A.nidulans            EHEVDANGKSRYTLGARAEPEDDE------------------------------------    990
Z.triciti             KHERDENGDARWTLATKATDDEDDD-----------------------------------    1007
                       ** .

S.cerevisiae          -------------------------------------------------------- 918
V.dahliae             -------------------------------------------------------- 886
M.oryzae              -------------------------------------------------------- 926
N.crassa              -------------------------------------------------------- 920
T.reesei              -------------------------------------------------------- 923
F.graminearum         -------------------------------------------------------- 922
F.oxysporum           -------------------------------------------------------- 923
F.verticillioides     -------------------------------------------------------- 923
C.neoformans          GGKKGATGGDKGLSDQTGKGHEHAQAQGKGANEVQPDGTQPKPDDQSSEGTHVDPN 1079
A.nidulans            -------------------------------------------------------- 990
Z.triciti             -------------------------------------------------------- 1007
```

Supplementary Figure S5. Amino acid sequence alignment of Pma1 proteins from *S. cerevisiae* (YGL008C), *V. dahliae* (XP_009652327), *M. oryzae* (MGG_07200), *N. crassa* (NCU01680), *T. reesei* (TRIREDRAFT_78757), *F. graminearum* (FGRAMPH1_01G03505), *F. oxysporum* (FOXG_11289), *F. verticillioides* (FVEG_09502), *C. neoformans* (CNAG_03565), *A. nidulans* (AN4859) and *Z. triciti* (ZTRI_5.411). Boxed in red is the Ser-507 in *S. cerevisiae* Pma1 and the corresponding Thr residue conserved in filamentous fungi. Boxed in green is the consensus phosphorylation motif for Ck1.
